# Aging Does Not Affect Beta Modulation during Reaching Movements

**DOI:** 10.1155/2019/1619290

**Published:** 2019-05-15

**Authors:** Serena Ricci, Ramtin Mehraram, Elisa Tatti, Aaron B. Nelson, Martina Bossini-Baroggi, Priya Panday, Nancy Lin, M. Felice Ghilardi

**Affiliations:** ^1^CUNY Medical School, New York, NY 10031, USA; ^2^DIBRIS University of Genoa, 16145, Italy; ^3^Institute of Neuroscience, Newcastle University, NE17RU, UK

## Abstract

During movement, modulation of beta power occurs over the sensorimotor areas, with a decrease just before its start (event-related desynchronization, ERD) and a rebound after its end (event-related synchronization, ERS). We have recently found that the depth of ERD-to-ERS modulation increases during practice in a reaching task and the following day decreases to baseline levels. Importantly, the magnitude of the beta modulation increase during practice is highly correlated with the retention of motor skill tested the following day. Together with other evidence, this suggests that the increase of practice-related modulation depth may be the expression of sensorimotor cortex's plasticity. Here, we determine whether the practice-related increase of beta modulation depth is equally present in a group of younger and a group of older subjects during the performance of a 30-minute block of reaching movements. We focused our analyses on two regions of interest (ROIs): the left sensorimotor and the frontal region. Performance indices were significantly different in the two groups, with the movements of older subjects being slower and less accurate. Importantly, both groups presented a similar increase of the practice-related beta modulation depth in both ROIs in the course of the task. Peak latency analysis revealed a progressive delay of the ERS peak that correlated with the total movement time. Altogether, these findings support the notion that the depth of beta modulation in a reaching movement task does not depend on age and confirm previous findings that only ERS peak latency but not ERS magnitude is related to performance indices.

## 1. Introduction

Movement is associated with changes of the electroencephalographic (EEG) activity in the beta frequency range from 15 to 30 Hz recorded mainly over the sensorimotor cortex. During movement planning, beta power decreases reaching a minimum at the end of the movement, event-related desynchronization (ERD) [1]. After the movement, beta power shows a rebound, defined as event-related synchronization (ERS) [2]. Likely, ERD reflects the increased excitability of the motor cortex and the deactivation of somatosensory areas. ERS, instead, may represent the reactivation of the somatosensory area following the motor activity [3, 4]. There is no clear evidence as to whether ERD and ERS characteristics are related to specific movement attributes [5–7] or whether they change with aging or neurodegenerative processes [8–10]. Interestingly, we have recently found that during practice in a reaching task, ERS magnitude increases [11, 12], independently of possible changes in mean power. Such practice-related increases are also evident in the beta modulation depth, computed as the ERS-ERD peak-to-peak difference. Importantly, we have also found that beta modulation decreased to baseline levels twenty-four hours later and that the magnitude of its increase during practice was correlated with retention of motor skill tested the following day [10]. Thus, we interpreted the beta modulation changes occurring during practice as reflecting plasticity-related phenomena: indeed, human and animal studies have shown that beta power increases in parallel with a reduction of cortical excitability and with an increase of GABA levels [13, 14]. In this context, the recurring activation and inactivation of the sensory and motor areas during our task with repetitive reaching movements may be an appropriate scenario to trigger long-term potentiation- (LTP-) related phenomena and may result in an increase of beta modulation depth. In turn, such an increase may reflect a progressive saturation of the mechanisms related to LTP-like plasticity. This idea is supported by work in animal and humans. In particular, high beta power likely reflects high GABA levels [14–20], thus linking increases in beta power to increases of inhibitory processes as well as to decreases of cortical excitability and LTP-related processes. Furthermore, theta burst TMS protocols that modulate local plasticity also induce local changes not only in cortical excitability but also in beta power [13, 21, 22]. Finally, in Parkinson's disease (PD), which is characterized by alterations in the beta frequency range [23–25] and impaired plasticity [26–31], we did not find either the practice-related increases of beta power or the retention of motor improvement that was present in normal subjects. As neural plasticity declines with increasing age [32], it is possible that practice-related beta modulation would also be affected by aging. However, studies on either beta oscillatory activity or plasticity of the sensorimotor cortex report controversial results. For instance, a recent work with a motor sequence task reported increased ERD amplitude over the contralateral sensorimotor area in older (aged from 54 to 75 years) compared to younger adults (aged from 20 to 42 years) [8]. In contrast, another study with a grip task [19] showed a lack of correlation between age and movement-related beta ERD in the contralateral motor area. Similarly, the studies testing LTP-like plasticity of the sensorimotor cortex with paired-associative stimulation (PAS) protocols yielded contrasting results [33–35].

In the present study, we ascertain in a group of younger and older adults whether healthy aging affects practice-related changes both in terms of magnitude and peak latency of ERD and ERS during a reaching task with the right hand. We focused on the left sensorimotor cortex and on a frontal region that, in previous studies, showed robust beta modulation [11, 12].

## 2. Materials and Methods

### 2.1. Subjects

We tested two groups of subjects: a younger group, with thirteen subjects (mean age ± SD: 24.2 ± 4.5 years, ten women) and an older group with thirteen subjects (mean age ± SD: 57.5 ± 8.2 years, six women). All subjects were right-handed, as determined by the Edinburgh inventory [36], and had no history of neurological or psychiatric disorders. Experiments were conducted with the approval of our Institutional Review Board, and written informed consent was obtained from all participants.

### 2.2. Experimental Design and Motor Task

**Figure 1:**
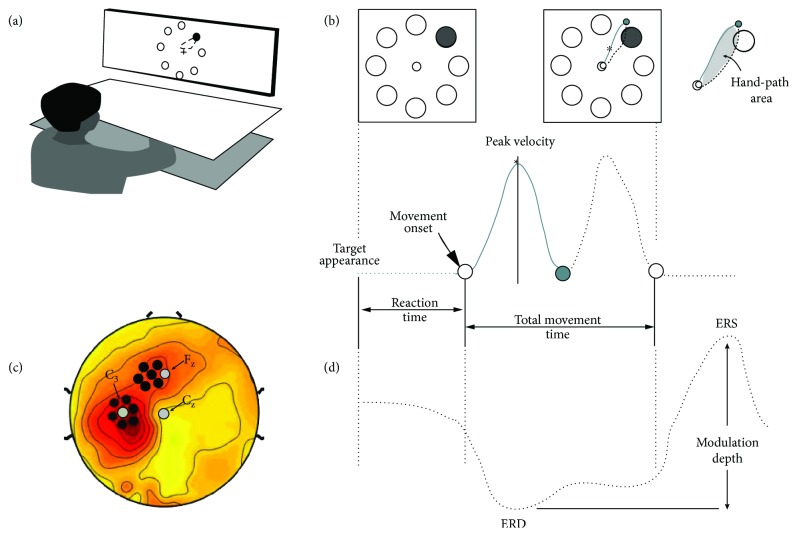
Experimental design and kinematic measures. (a) Testing set up. (b) Upper line: target display on the screen with the appearance of the target (left); the hand-path display during the movement (center); in grey, the hand-path area (left). Bottom line: temporal profile of trajectory velocity with the kinematic parameters used in the analyses. (c) Definition of the regions of interest (ROIs): the left and the frontal ROIs (black dots) with Cz, Fz, and C3 (grey dots). (d) Representation of beta power changes related to target appearance, movement onset, and end. The peak of event-related desynchronization (ERD) is followed by a rebound (or event-related synchronization, ERS) after the movement end. Beta modulation is defined as the difference between ERD and ERS power.

All experiments were run in the morning. Subjects were fitted with high-density 256-electrode EEG cap. The two groups of subjects performed a reaching task (MOT) for 30 minutes. Specifically, as described in previous papers [37, 38], subjects were seated in front of a computer screen and moved a hand-held cursor on a digitizing tablet with their right upper limb that was hidden by an opaque surface (Figure 1(a)). On the screen, an array of eight targets (1 cm diameter circles) that was equidistant (4 cm) from a central starting point was present at all times together with the position of the cursor. One of the eight targets blackened with a 1.5 s interval for 400 ms in a random order; subjects were instructed to move as soon as possible, minimizing reaction time but avoiding anticipation or guessing, to make overlapping out-and-back movements without corrections or stops with sharp reversal within the target (Figure 1(b)). Before the first testing session, all subjects were trained in this task to reach a hit rate of at least 95%.

The session encompassed a total of 840 target presentations divided into 15 sets of 56 each. Between two consecutive sets, subjects paused for an average of 30 seconds.

For each movement, we computed the following: reaction time, defined as the time from the target appearance to the movement onset; total movement time, defined as the time from movement onset to the end of the out and back stroke; peak velocity; and hand-path area, a measure that reflects interjoint coordination, computed as the area included in the trajectory normalized by path length squared (Figure 1(b)). For each subject, we discarded outlier movements that met one of the following criteria: reaction time exceeding 2 SD from the subject's mean, directional error greater than 22°, and movement end less than 100 ms before the presentation of the next target.

### 2.3. EEG

#### 2.3.1. EEG Recording

A high-density EEG was recorded from 256 electrodes (HydroCel net, Electrical Geodesics Inc.) for the entire duration of the experiment with a sampling rate of 250 Hz, using the high impedance amplifier Net Amp 300 and Net Station 4.3. Impedances were kept below 30 kΩ, and the signal was referenced to the vertex Cz.

#### 2.3.2. EEG Data Preprocessing

Data were preprocessed using the public Matlab toolbox EEGLAB [39]. The continuous EEG signal was filtered with a passband two-way least-square FIR filter between 1 and 80 Hz and a notch filter centered at 60 Hz. Then, signal was divided into 4-second epochs, centered at the stimulus onset (-1 to 3 seconds). Also, recordings were visually inspected to define artefactual epochs and channels. Channels affected by bad scalp-electrode contact were replaced with spherical spline interpolation, and artefactual epochs were removed from the recording. Stereotypical artifacts, such as blinks, eye movements, and motion-related signals, were removed with Independent Component Analysis (ICA) with Principal Component Analysis- (PCA-) based dimension reduction [40]. Briefly, we visually inspected the power spectral density, topographical maps, and time activations of each estimated component. The components identified as “artefactual” were removed from the raw EEG signal. Finally, the signal was averaged referenced to proceed with the analyses and reduced to 180 channels, removing 76 channels located on the cheeks and the neck.

#### 2.3.3. EEG Data Analyses

As a first step, we aligned each valid trial (i.e., trials that were not discarded from either EEG or kinematic preprocessing) to the time of movement onset; then, we computed time-frequency representations in the 15 to 30 Hz range (0.25 Hz steps), using a short-time Fourier transform approach (Hanning taper, time step-size of 20 ms, 5 cycles adaptive window width). Beta power of each trial and region of interest (ROI) was normalized using the average beta power value computed over the entire motor session as such: ∑_*i*_^*i*=1:trials^((BetaEEG*i* − TotPower)/TotPower), with TotPower defined as the total beta power across all the trials. First, we ascertained whether the two groups of subjects had a comparable beta power in both ROIs with nonparametric permutation testing with false discovery rate correction. Then, we determined the amplitudes of ERD and ERS: ERD amplitude was defined as the minimum value of beta power within an interval between 200 ms before movement onset to 700 ms after it; ERS amplitude was the maximum value in the interval from 500 to 1500 ms. Beta modulation depth was computed as the difference between ERS and ERD. We then averaged the data of all the subjects to define two ROIs centered where beta modulation was maximal. Specifically, we defined a left sensorimotor ROI, which was centered on C3 with six neighbor electrodes, and a frontal ROI, which was centered on the electrode between Fz and F3 and its six neighbors (Figure 1(c)).

Afterwards, trials were averaged across sets to determine time and group differences, as well as across all trials to assess time differences in the two groups. All the analyses have been implemented using the FieldTrip toolbox for Matlab [41].

### 2.4. Statistical Analysis

To quantify the changes of EEG (ERD and ERS magnitude and peak latency) and kinematic measures (reaction time, peak velocity, hand-path area, and total movement time) across sets, we performed repeated measure multivariate ANOVAs (MANOVA) with Group (younger, older) as between-subject effect and practice (15 sets) and parameters and ROI (left and front, only for the EEG analysis) as within-subject effects. We also used univariate mixed model ANOVA for EEG parameters for each ROI (including beta modulation depth) and for kinematic measurements with practice (15 sets) as within-subject effect. All the results had been Greenhouse-Geisser corrected since the assumption of sphericity was violated (Mauchly's test). Pearson coefficients were used to explore significant correlations between kinematic and EEG peak latency parameters. Normality was tested with the Lilliefors test. We used two-tailed paired *t*-tests to determine a significant group difference for both ERD and ERS peak latency. Results were considered significant with a *p* value < 0.05. All statistical analyses were conducted using SPSS v25 and Matlab 2017b.

## 3. Results and Discussion

### 3.1. Motor Performance Differs in Younger and Older Subjects

**Figure 2:**
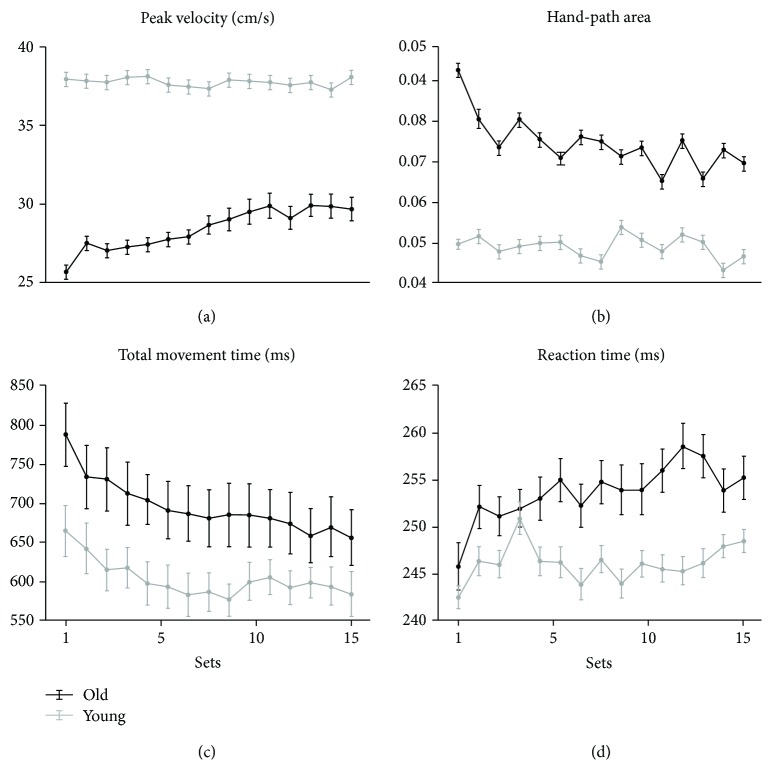
Average of kinematic measures of each set (56 movements each) for the younger (grey lines and dots) and older (black lines and dots) groups. The vertical bars represent standard errors of the mean.

All participants completed the session without any difficulty. In general, movements were mostly straight with bell-shaped velocity profiles in all subjects. The performance measures of the two groups across sets are illustrated in Figure 2. The results of the repeated measure MANOVA revealed an overall effect of practice (*F*_(56, 1297.47)_ = 2.86, *p* < 0.001) and group (*F*_(4, 21)_ = 8.80, *p* < 0.001) and a trend toward significance in the Practice∗Group interaction (*F*_(56, 1297.47)_ = 1.26, *p* = 0.096). Results of the univariate tests are reported in Table 1. Specifically, peak velocity values were significantly greater in the younger group without significant changes across sets (Table 1, Figure 2(a)). However, inspection of the data showed some increase across sets, although not significant, only in the older group (Figure 2(a)). In parallel, total movement time was significantly longer in older subjects, with significant decreases across sets in both groups (Table 1, Figure 2(c)). Hand-path area, an inverse measure of spatial accuracy that depends on velocity, was greater in the older participants but decreased in both groups across sets, as shown by a borderline *p* value (Table 1, Figure 2(b)). Finally, reaction times were rather stable across sets and were slightly longer in the older group, despite mixed model ANOVA did not reveal any main effect or interaction (Table 1, Figure 2(d)). In summary, these results suggest that movements in the older group were slower and spatially less accurate than in the younger group, as confirmed by correlations between the mean values of kinematic parameters and age (peak velocity: *R* ^2^ = 0.43, *p* < 0.001; hand-path area: *R* ^2^ = 0.28, *p* = 0.006; total movement time: *R* ^2^ = 0.30, *p* = 0.004). Additionally, we found a strong correlation between age and the decrease of hand-path area from the first to the last set (*R* ^2^ = 0.29, *p* = 0.005, Supplementary Table 1). Deterioration of motor performance in the elderly has been reported in several publications, involving spatial and temporal characteristics of motor performance. The causes of this decline are multiple and may involve, singly or in combination, muscular, skeletal, and the central and peripheral nervous systems. During aging, progressive muscle deterioration [42], increased muscle fatigability, and sarcopenia [43–46] may occur with loss of motor units, remodeling of neuromuscular junctions, and eventually with alteration of peripheral and neuromuscular transmissions [47–49]. Besides decreasing muscle strength [50], aging may also impair proprioceptive processing [51], as well as the function of cortical motor regions [48] and the basal ganglia [52]. This can result in mobility problems and an increased risk of falling [53]. Our results showed that, besides being slower, the movements of the older group had greater hand-path area values, suggesting a worse interjoint coordination [54–56]. This occurred despite the older groups' movements were slower, and thus, the interaction torques developing during movement should have been easier to counteract, and movements should have had overlapping trajectories. Intact proprioception is necessary for overcoming these forces not only by providing feedback during the movement but also by updating the sensorimotor memories used to program movements through feedforward mechanisms [54–56]. Therefore, even small problems in proprioception information processing, as the ones reported in aging, may produce deficits of intersegmental dynamics, despite low movement velocities, thus resulting in increases of both interjoint timing and hand-path area. Importantly, the older group also showed a decrease of hand-path area across sets with values approaching the range of the younger group in the last sets. This decrease occurred together with an increase of peak velocity, suggesting that some skill learning occurred in the older group. These practice-related improvements also indicate a shift toward the use of feedforward mechanisms and possibly suggest memory formation in this particular aspect of performance [11]. To be noticed, performance of the younger group reached a plateau already in the first set, thus minimizing the significance of the improvements during the last set. This conclusion is supported also by the fact that the values of hand-path area in the older group hardly reached those of the younger subjects (see Figure 2(b)). Therefore, our results suggest that, despite average differences, both groups displayed some learning.

### 3.2. Beta Modulatory Activity Is Similar in Younger and Older Subjects

**Figure 3:**
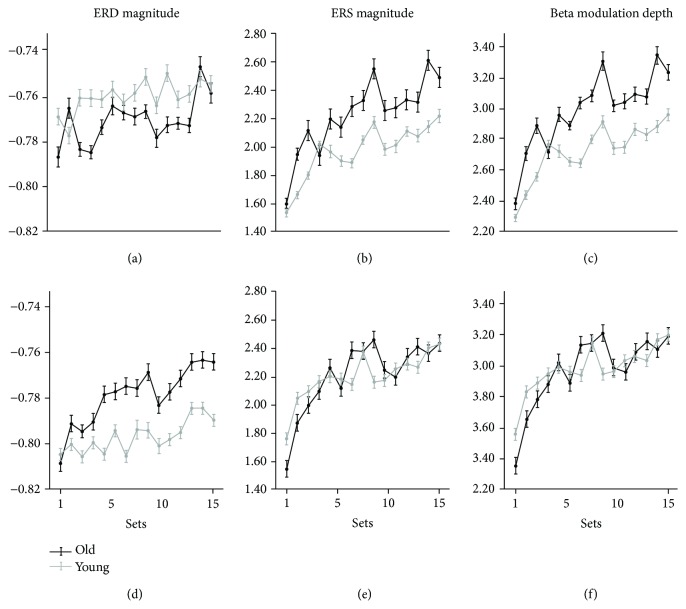
Average of ERD (a, d), ERS (b, e), and beta modulation (c, f) magnitudes for the left (upper row) and frontal (lower row) ROIs for each set in the younger (grey lines and dots) and older (black lines and dots) groups. The vertical bars represent the standard errors of the mean.

We next analyzed the power changes across sets of beta ERD and ERS in the two groups for the left and frontal ROIs. Results are reported in Table 2 and Supplementary Table 2 and illustrated in Figure 3. Multivariate tests with ERD and ERS magnitude did not reveal significant group differences or interactions (Group: *F*_(2, 23)_ = 1.121, *p* = 0.343; Practice∗Group: *F*_(28, 672)_ = 0.548, *p* = 0.973; ROI∗Group: *F*_(2, 23)_ = 1.47, *p* = 0.251; Practice∗ROI: *F*_(28,670)_ = 0.899, *p* = 0.618; Practice∗ROI∗Group: *F*_(28, 670)_ = 1.00, *p* = 0.465). Nevertheless, we found the main effects of practice and ROI (*F*_(28, 672)_ = 4.69, *p* < 0.001; *F*_(2, 23)_ = 3.71, *p* = 0.040, respectively). The results of univariate mixed model ANOVAs for ERD and ERS (Table 2) confirmed a significant effect of practice for both measures. They also revealed a difference between ROIs for ERD magnitude (see also: Supplementary Table 2 and Figure 3). Over the frontal ROI, the significant across-set increase extended also to beta ERD (Supplementary Table 2): inspection of the data in Figure 3 suggests that such an effect was more evident, although not significantly so, in the older group. Indeed, some works have shown that older participants need to recruit additional resources in sensorimotor and premotor areas to achieve normal movement execution [57–61]. Thus, the progressive increase of ERD magnitude in older subjects over the frontal ROI may reflect an increased recruitment to improve performance across sets. Univariate mixed model ANOVA for beta modulation depth (Table 2) revealed only a main effect of practice, without any interaction between variables. Finally, as in previous studies [11, 12], we found no significant correlation between the practice-related changes of ERD, ERS, or beta modulation and the performance changes across sets. Altogether, these results suggest that practice-related changes of beta modulation in both ROIs are very similar in the two groups. Further analyses on all subjects showed no correlation between the mean values of ERD, ERS, and modulation depth and the subjects' age for both left and frontal ROIs (all: *R* ^2^ < 0.01, *p* > 0.50). Also, correlations between magnitude difference across sets and age did not reveal any direct link between beta modulation and anagraphical age (Supplementary Table 1). These results are in agreement with a study showing no age effect on the mean value of ERD recorded over the left sensorimotor area with a grip task [19], but at odds with a work demonstrating that mean ERD amplitude was greater in older subjects during a motor sequence task [8]. It is conceivable that such discrepant results may originate from differences in task characteristics and sample size. Also, anagraphical age may not be the only and best predictor of changes in cortical function and performance, as it is increasingly more evident that exercise and other factors play important roles in decreasing or accelerating aging processes.

As mentioned earlier, the most novel result is that the increases of beta modulation across sets are similar in the younger and older groups, and notably, this occurs despite important group differences in performance indices (see Figure 2). This result prompts two sets of considerations. First, the magnitude of movement-related beta oscillations does not directly reflect movement characteristics, in line with other studies reporting a lack of correlation between ERD ERS and speed [5, 62], force [63–65], movement type [6], or muscle pattern [7]. Specifically, previous studies found beta oscillatory differences in slow versus fast movements [5]; however, a correlation between EMG burst and ERS latency was detected only for slow movements. Similarly, no direct relationship between movement parameters and ERS, despite an ERS difference between extension-flexion and flexion-extension movements, had been found [66]. Also, a recent work from our research group indicated that no link between movement extent and beta modulation magnitude is detected in upper limb reaching movements [67]. Indeed, movement-related beta oscillations may be related to sensorimotor integration processes associated with movement planning and execution rather than explicitly reflecting the coding of distinct movement features [68, 69]. The second set of considerations is based on the fact that the continuous performance in our motor tasks should induce constant and regular interplay of sensory and motor regions' activities [68, 69], thus providing the bases for use-dependent LTP induction. Improvements of velocity and interjoint coordination indices during the task in the older group indicate a major shift of the performance toward a reinforcement of the feedforward mechanisms, and thus of memory formation. Practice-related beta modulation increase may reflect this phenomenon [11]. If indeed, as also suggested by other evidence [13, 14], beta modulation depth increases reflect LTP-like phenomena in the sensorimotor cortex; then, one may speculate that plasticity-related mechanisms in the sensorimotor cortex should not be particularly affected by age. Studies testing the effect of age on the plasticity of the sensorimotor cortex with transcranial magnetic stimulation (TMS) have not shown clear differences between younger and older subjects, and the picture may be further complicated by the influence of hormonal levels on PAS results [33, 70] and neural plasticity in general [71, 72]. Indeed, our results need to be replicated in a larger population, also taking into account the factors other than age that could affect cortical plasticity mechanisms, such as motor and cognitive reserves.

### 3.3. ERS Peak Latency Occurs Later in Older Adults and Correlates with Total Movement Time

**Figure 4:**
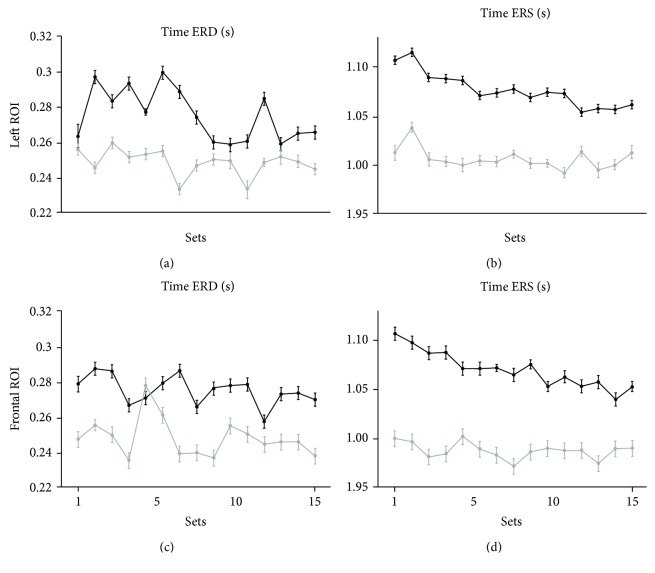
Average peak latencies of ERD (a, c) and ERS (b, d) for each set in the younger (grey circles and lines) and older (black circles and lines) groups for the left (upper graphs, a and b) and frontal (lower graphs, c and d) ROIs. The vertical bars represent the standard error of the mean.

**Figure 5:**
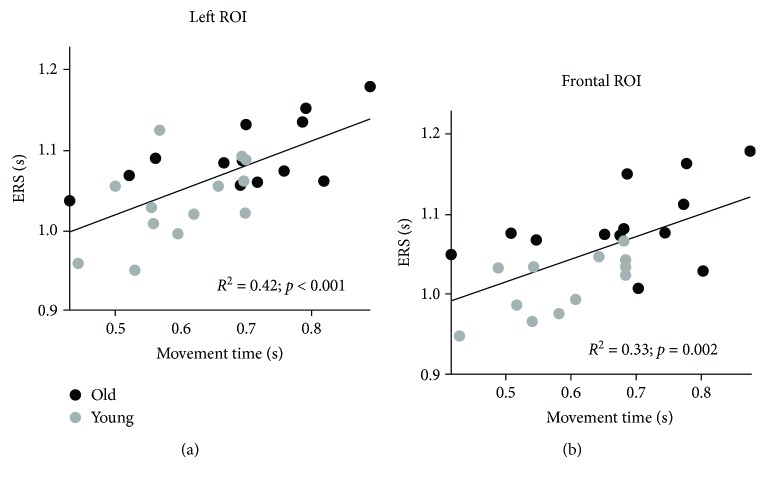
Correlations of total movement time with ERS peak latency in the left (a) and frontal (b) ROIs for younger (gray points) and older (black points) subjects combined.

We finally focused on the peak latency of ERD and ERS peaks and determined whether they changed across sets and groups. Indeed, inspection of the data suggests that ERS peak latency was higher in the older but decreased with practice in both groups (Figure 4). Statistical analyses (Table 3, Supplementary Table 3) showed a significant group difference (*F*_(2, 23)_ = 7.93, *p* = 0.002) and a significant effect of practice (*F*_(28, 670)_ = 1.75, *p* = 0.010). However, we found no significant effect of ROI (*F*_(2, 23)_ = 2.30, *p* = 0.123) and interactions (ROI∗Group: *F*_(2, 23)_ = 0.634, *p* = 0.540; Practice∗Group: *F*_(28,670)_ = 0.95, *p* = 0.535; Practice∗ROI: *F*_(28,670)_ = 0.84, *p* = 0.705; and Practice∗ROI∗Group: *F*_(28, 670)_ = 0.80, *p* = 0.766). Importantly, the temporal occurrence of ERS peak was linked to total movement time in both the left and the frontal ROIs (*R* ^2^ = 0.42, *p* < 0.0001; *R* ^2^ = 0.33, *p* = 0.002, respectively; Figure 5). However, ERS peak latency did not correlate with other kinematic parameters and with either ERS amplitude or beta modulation depth in both the left and the frontal ROI (*R* ^2^ < 0.08, *p* > 0.16). Altogether, these results show no effect of aging on ERS and ERD peak latency but only a strong dependence of ERS peak latency on movement duration. This is further supported by the lack of significant correlation between changes in peak latency across sets and age (Supplementary Table 1).

Only a few studies have focused on the ERS peak latency or its duration. It is generally accepted that ERS occurs 300-1000 ms after movement ends and lasts several seconds [2, 5, 63, 73–75]. Indeed, the present study with fast reaching movements at a pace of 1.5 s in a choice reaction time task showed that, on average, the peak latency of peak ERS is highly correlated with the total movement time and that it occurs from 300 to 400 ms from the end of the out-and-back movement. This observation is in agreement with the idea that ERS peak coincides with a deactivated state of the motor cortex and thus to a reduced excitability of the neuronal populations [76]. The peak latency characteristics of ERS are linked to the type of task and the movement duration. In tasks with isometric wrist contractions, ERS occurrence is related to the rate of force development but not the force output [63, 64]. During a task with repetitive movements, ERS occurs earlier than in a task with discrete finger movements [65]. Another work demonstrated that ERS lasts longer after withholding of real foot movements compared to imagined foot movements [77], with the faster movements showing earlier ERS peak occurrence.

## 4. Conclusions

The main result of this study is that, despite being slower and less accurate, the reaching movements of older subjects are associated with beta oscillatory activity that is no different from that accompanying the faster and more precise performance of younger subjects. Importantly, in both groups, the magnitude of beta modulation depth increases to the same degree during practice in both the left and the frontal ROIs. To address the discrepancy between performance and EEG, the results of this study need to be replicated in a larger population and also to take into account the factors other than anagraphical age.

## Figures and Tables

**Table 1:** Results of the repeated measure ANOVA, univariate tests of peak velocity, hand-path area, and reaction time comparing groups across practice.

	Group		Practice		GroupXPractice	
	*F*_(1, 24)_	*p*	*F*_(14,336)_	*p*	*F*_(14,336)_	*p*
Peak velocity	**9.51**	**0.005**	1.32	0.277	1.63	0.209
Hand-path area	**8.63**	**0.007**	2.02	0.059	1.80	0.095
Reaction time	0.47	0.501	1.52	0.189	0.92	0.468
Total movement time	**4.82**	**0.038**	**14.94**	**<0.001**	0.78	0.515

**Table 2:** Results of mixed model univariate ANOVAs for the magnitude of ERD and ERS and beta modulation depth.

	Group (G)		Practice (P)		ROI (R)		P∗G		R∗G		P∗R		P∗R∗G	
	*F*	*p*	*F*	*p*	*F*	*p*	*F*	*p*	*F*	*p*	*F*	*p*	*F*	*p*
ERD	0.07	0.788	**2.48**	**0.035**	**6.88**	**0.015**	0.51	0.770	3.05	0.094	1.01	0.424	1.39	0.225
ERS	0.68	0.417	**8.97**	**<0.001**	1.09	0.307	0.67	0.654	1.59	0.219	0.69	0.626	0.83	0.526
*β* Modulation	0.56	0.460	**9.13**	**<0.001**	1.44	0.241	0.67	0.657	1.78	0.195	0.71	0.614	0.78	0.563

**Table 3:** Result of repeated measure ANOVAs for ERD and ERS peak latency.

	Group (G)		Practice (P)		ROI (R)		P∗G		R∗G		P∗R		P∗R∗G	
	*F*	*p*	*F*	*p*	*F*	*p*	*F*	*p*	*F*	*p*	*F*	*p*	*F*	*p*
ERD	**4.35**	**0.048**	1.07	0.386	0.00	0.959	0.78	0.649	0.00	0.975	1.03	0.419	0.93	0.501
ERS	**12.42**	**0.002**	**2.74**	**0.015**	3.96	0.058	1.13	0.350	1.14	0.296	0.65	0.747	0.66	0.730

## Data Availability

Data that support the findings of this study are available from the corresponding authors upon reasonable request.

## Supplementary Materials

Supplementary Materials Supplementary Table 1: correlations between Behavioral parameters and age. Supplementary Table 2: results of the Mixed Model ANOVA for ERD, ERS, and beta modulation comparing groups across practice in the two ROIs. Supplementary Table 3: results of the Mixed Model ANOVA for ERD, ERS, and beta modulation peak latency.
