# Supplementary material for: Aging Does Not Affect Beta Modulation during Reaching Movements
Source: Neural Plast. 2019 May 15;2019:1619290. doi: 10.1155/2019/1619290 (PMC6541950; doi:10.1155/2019/1619290)
Supplement: Supplementary Materials — Supplementary Table 1: correlations between Behavioral parameters and age. Supplementary Table 2: results of the Mixed Model ANOVA for ERD, ERS, and beta modulation comparing groups across practice in the two ROIs. Supplementary Table 3: results of the Mixed Model ANOVA for ERD, ERS, and beta modulation peak latency. [file 1619290.f1.pdf]

Supplementary Materials

Supplementary Table 1: Correlations between Behavioral parameters and age

ΔINDEX VS AGE	**R^2**	**P**
Reaction time	0.00	0.853
Peak velocity	0.02	0.490
**Hand path area**	**0.29**	**0.005**
Total movement time	0.04	0.330
	Left ROI	
ERD magnitude	0.03	0.384
ERS magnitude	0.05	0.286
ERD peak latency	0.01	0.722
ERS peak latency	0.08	0.177
	Frontal ROI	
ERD magnitude	0.12	0.085
ERS magnitude	0.03	0.400
ERD peak latency	0.01	0.612
ERS peak latency	0.09	0.143

Supplementary Table 2: Results of the Mixed Model ANOVA for ERD, ERS, and beta modulation comparing groups across practice in the two ROIs.

	**Group**		**Practice**		**GroupXPractice**	
	**F**	**P**	**F**	**P**	**F**	**P**
			Left ROI			
ERD	0.52	0.477	1.36	0.246	0.65	0.664
ERS	1.76	0.197	**7.71**	**<0.001**	0.63	0.666
Beta Modulation	1.72	0.203	**7.95**	**<0.001**	0.68	0.661
			Frontal ROI			
ERD	1.09	0.308	**3.43**	**<0.001**	0.75	0.591
ERS	0.00	0.959	**6.19**	**<0.001**	0.75	0.571
Beta Modulation	0.00	0.962	**6.05**	**<0.001**	0.72	0.595

Supplementary Table 3: Results of the Mixed Model ANOVA for ERD, ERS, and beta modulation peak latency.

	**Group**		**Practice**		**GroupXPractice**	
	**F**	**P**	**F**	**P**	**F**	**P**
			Left ROI			
ERD	3.99	0.057	1.20	0.296	1.07	0.388
ERS	**9.78**	**0.005**	**2.46**	**0.023**	0.82	0.562
			Frontal ROI			
ERD	4.20	0.052	0.91	0.516	0.61	0.777
ERS	**13.56**	**0.001**	1.82	0.084	1.16	0.330
